# Effectiveness of pelvic floor rehabilitation after radical prostatectomy and continence recovery in relation to surgical technique

**DOI:** 10.1038/s41598-026-36972-7

**Published:** 2026-03-06

**Authors:** Małgorzata Terek-Derszniak, Danuta Gąsior-Perczak, Małgorzata Biskup, Tomasz Skowronek, Mariusz Nowak, Justyna Falana, Jarosław Jaskulski, Mateusz Obarzanowski, Stanislaw Gozdz, Pawel Macek

**Affiliations:** 1 Department of Rehabilitation, Holycross Cancer Centre, Kielce, Poland; 2 Collegium Medicum Jan Kochanowski University of Kielce, Kielce, Poland; 3 Oncology Clinic, Holycross Cancer Centre, Kielce, Poland; 4 Department of Urology, Holycross Cancer Centre, Kielce, Poland; 5 Endocrinology Clinic, Holycross Cancer Centre, Kielce, Poland; 6 Scientific Research, Epidemiology and R&D Centre, Holycross Cancer Centre, Kielce, Poland

**Keywords:** Urinary incontinence, Prostatectomy, Pelvic floor, Robot-assisted surgery, Laparoscopic surgery, Prognostic factors, Diseases, Medical research, Oncology, Urology

## Abstract

The potential influence of surgical technique and pelvic floor rehabilitation on continence recovery continues to be debated. This prospective cohort study included 182 men undergoing robot-assisted or laparoscopic radical prostatectomy. All patients were referred for physiotherapist-guided pelvic floor muscle training. Urinary incontinence was assessed using a standardized 1-h pad test and a three-stage UI grading system at 1, 3, and 6 months after catheter removal. Ordinal and logistic regression models were used to identify predictors of continence, including surgical technique, age, pathological features, and rehabilitation-related variables. Exploratory time-to-event analyses (Cox proportional hazards models) were also performed to assess the dynamics of continence recovery, with catheter removal as time zero and achievement of full continence (pad test ≤ 2 g) as the event. Patients undergoing RARP demonstrated higher odds of early full continence shortly after surgery (OR = 5.26; 95% CI 2.34–11.80; *p* < 0.001) and functional continence (OR = 4.00; 95% CI 1.96–8.14; *p* < 0.001). After adjustment for baseline incontinence severity, surgical technique was no longer an independent predictor of continence recovery. However, in multivariable analyses, baseline incontinence severity and timing of rehabilitation were the strongest independent predictors of continence recovery. Robotic prostatectomy is associated with a more favorable continence status at the start of rehabilitation, whereas continence recovery during physiotherapy is primarily determined by baseline incontinence severity and timely initiation of pelvic floor therapy rather than surgical approach. These results highlight the need to integrate precise surgical techniques with standardized, early, and individualized physiotherapy to optimize continence recovery and improving quality of life after radical prostatectomy.

**Supplementary Information:**

The online version contains supplementary material available at 10.1038/s41598-026-36972-7.

## Introduction

Robot-assisted radical prostatectomy (RARP) is a widely accepted surgical approach for the treatment of localized prostate cancer ^1^.Compared to other techniques, RARP offers equivalent oncological outcomes while providing superior functional results ^2^.The enhanced visualization of the operative field and improved preservation of anatomical structures afforded by robotic systems contribute to lower rates of postoperative complications ^1^. However, despite these advantages, patients undergoing RARP may still experience both short- and long-term complications, including urinary incontinence and sexual dysfunction ^3^. Postoperative urinary incontinence (UI) has a significant negative impact on quality of life, often leading to social withdrawal, embarrassment, and psychological distress ^4,5^. Although in many patients UI is temporary, a subset experiences persistent symptoms ^6^. According to the European Association of Urology (EAU) guidelines, initial management of UI should involve non-invasive measures, beginning with conservative therapy with or without pharmacological support followed by surgical interventions if conservative treatment fails ^7–9^.

Pelvic floor muscle training (PFMT) is the first-line conservative treatment recommended for patients with UI after radical prostatectomy. Its effectiveness has been demonstrated in numerous studies; however, individual response may depend on the baseline severity of incontinence and the surgical approach used ^10–12^. Current evidence regarding the relationship between surgical technique and UI remains inconclusive. While a recent meta-analysis (2023) suggested that RARP may lead to better functional outcomes compared to laparoscopic radical prostatectomy (LRP) ^1^, earlier studies, such as those by Gershman et al., reported no significant differences in functional results between surgical techniques ^1,13^. However, few studies have directly compared rehabilitation trajectories between patients treated with RARP and LRP, and none have clearly determined whether the surgical approach modifies the response to pelvic floor therapy. Most research has focused on the association between surgery and UI incidence, with limited attention to how surgical technique may affect the rehabilitation process. To date, it remains unclear whether patients undergoing RARP achieve better rehabilitation outcomes than those treated with LRP. Understanding whether surgical technique influences not only early continence but also the efficacy of rehabilitation is essential for clinical practice. If patients undergoing RARP benefit from a more favorable starting point, tailored rehabilitation protocols might not be necessary. Conversely, if the surgical method affects the trajectory of continence recovery, this could justify developing surgery-specific rehabilitation pathways. Moreover, there is insufficient evidence on whether the adoption of newer surgical approaches should be accompanied by updated rehabilitation recommendations.

Therefore, the aim of this study was to assess the impact of surgical approach (robot-assisted vs laparoscopic radical prostatectomy) on early urinary continence and the outcomes of pelvic floor muscle training.

## Materials and methods

### Study design and participants

This prospective cohort study was conducted between January 1, 2023, and January 31, 2025, at Holycross Cancer Center, Kielce, Poland. A total of 182 men scheduled to undergo radical prostatectomy (RP) for prostate cancer were enrolled. Of these, 106 underwent laparoscopic radical prostatectomy (LRP), and 76 underwent robot-assisted radical prostatectomy (RARP). The mean age of the cohort was 66.1 years (SD 6.5). All participants were referred to a physiotherapist approximately one month before surgery.

Inclusion criteria were adult men (≥ 18 years) undergoing RP for localized prostate cancer, with no prior neurological or urological disorders affecting urinary continence, and who provided written informed consent to participate. Exclusion criteria included inability to participate in physiotherapy due to medical contraindications, refusal to participate, or incomplete clinical data.

### Surgical procedures

All procedures were performed by two experienced robotic and five laparoscopic urologic surgeons, including two operators proficient in both techniques. Each surgeon had performed more than 100 radical prostatectomies before the study period, and no operations were conducted during the learning curve. The robotic program was initiated in 2023. The choice of surgical approach (robot-assisted radical prostatectomy, RARP, or laparoscopic radical prostatectomy, LRP) was determined by surgeon preference, system availability, and patient-specific clinical or anatomical factors such as tumor stage and prostate size. No Retzius-sparing procedures were performed. Bladder-neck-sparing was applied in approximately 90% of RARP and 60% of LRP cases. Nerve-sparing dissection was bilateral in about 60% of patients, unilateral in 20%, and omitted in 20%, with similar distributions across both surgical groups. Posterior reconstruction and dorsal venous complex (DVC) ligation were routinely performed in RARP, whereas in LRP, the DVC was ligated without posterior reconstruction. The duration of postoperative catheterization was similar in both groups (10–14 days). All patients received standard perioperative care, including antibiotic prophylaxis and early postoperative mobilization according to institutional protocol.

### Pelvic floor muscle training (PFMT)

The rehabilitation program consisted of four structured stages. Stage 0 (preoperative phase) was conducted approximately one month before surgery. Among the 182 participants, 146 patients attended three physiotherapist-supervised sessions, while 36 could not participate due to personal reasons. During this phase, patients were instructed in pelvic floor muscle localization, activation, and control using surface electromyography (sEMG; Noraxon Ultium) with an intra-anal probe and 50-mm surface electrodes (INTCO), as well as Medison Sono Ace PICO ultrasound (US).

### First session protocol:

- Patient instruction: Localization and voluntary activation of pelvic floor muscles.
- Examination position: Left lateral decubitus with hips and knees flexed. An intra-anal probe was inserted, and surface EMG electrodes were applied over the rectus abdominis and right gluteal muscle, following SENIAM (Surface Electromyography for the Non-Invasive Assessment of Muscles) guidelines.
- Exercise sequence (Glazer protocol) ^14^:
  1. Five fast contractions with immediate relaxation
  2. Five 10-s contractions with 5-s intervals
  3. One 30-s sustained contraction
  4. Probe and electrode removal
  5. Evaluation and feedback

During the two subsequent sessions, patients practiced controlled pelvic floor contractions in prone, sitting, and standing positions. Exercises included isolated pelvic floor activations, quick-release contractions, and breathing-synchronized maneuvers. Patients were instructed to perform these exercises four times daily, completing ten repetitions per set in different body positions, emphasizing standing and sitting. Additionally, each patient received a home exercise program including pelvic girdle and lower limb exercises and was advised to walk at least 30 min daily.

### Postoperative rehabilitation (stages I–III):

After catheter removal, patients resumed the same exercise regimen. Stage I rehabilitation began one month postoperatively. At the initial visit, a physiotherapist repeated the pelvic floor examination and performed a standardized 1-h pad test (PT; Seni Man Level 4 Extra Plus) to quantify urinary leakage. The pad was weighed before and after the test using a WLC6/F1/R medical scale (accuracy 0.1 g). Urinary incontinence (UI) was defined as urine loss > 2 g. During the test, patients performed the following activities:

- 15 min of drinking 0.5 L of water while seated
- 30 min of walking, including stairs
- 10 sit-to-stand repetitions
- 5 weight lifts from the floor
- 1 min jogging on the spot
- 10 coughs
- 1 min hand washing under running water

UI severity was classified based on pad test results as:

- Stage I: 2–10 g
- Stage II: 11–50 g
- Stage III: ≥ 50 g

The number of pads and other hygiene products (e.g., incontinence pads, adult diapers) was also recorded. Rehabilitation sessions were repeated at 1, 3, and 6 months after catheter removal. Patients with persistent leakage > 50 g or sensory deficits received adjunct neuromuscular electrostimulation, provided serum prostate-specific antigen (PSA) levels remained normal.

The following clinical variables, previously identified as predictors of UI, were included in the analysis: age, BMI, baseline UI stage, time from surgery to rehabilitation initiation, and pad test result ^9,15–17^.

### Outcome measures

Urinary continence was assessed at each follow-up visit using a standardized urinary incontinence (UI) staging system based on pad test results. Full continence was defined as UI stage 0, indicating complete dryness without the need for protective pads. Functional continence was defined as UI stage 0 or 1, corresponding to no or minimal urinary leakage (requiring at most one pad per day) without any meaningful impact on daily activities or quality of life. This distinction allowed clinically meaningful comparison of continence outcomes across rehabilitation stages and between surgical groups.

### Ethical approval

The study was approved by the Bioethics Committee of Collegium Medicum, Jan Kochanowski University, Kielce, Poland (approval no. 34/2018). All participants provided written informed consent before enrollment. The study was conducted in accordance with the Declaration of Helsinki.

### Statistical analysis

**Fig. 1:**
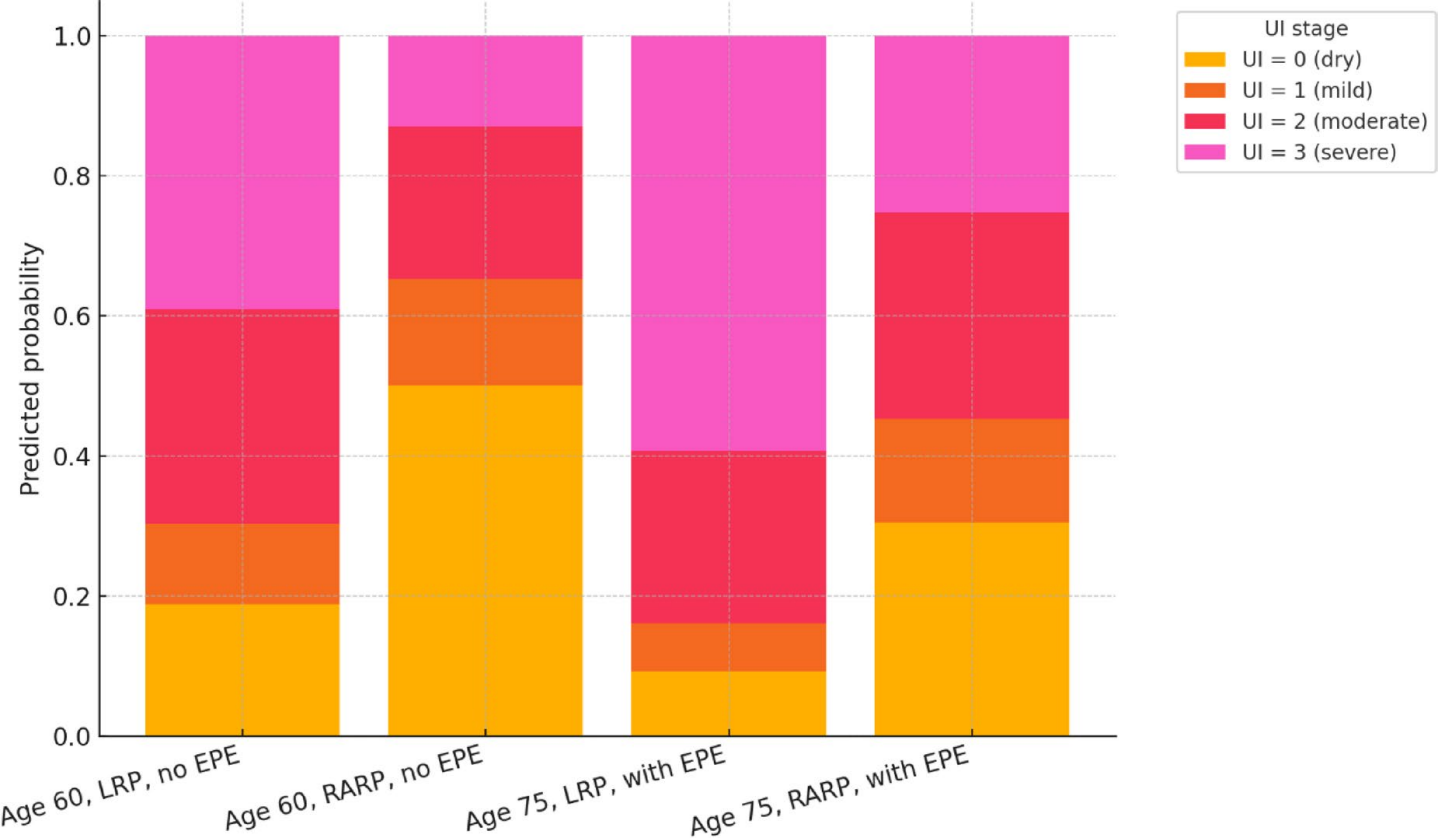
Predicted probabilities of urinary incontinence stages at baseline (Examination 1) according to patient age, surgical approach, and extraprostatic extension (EPE). *Note*: Stacked bar chart showing predicted probabilities of urinary incontinence (UI) stages at baseline (Examination 1), based on univariable ordinal regression models (Table S1). Scenarios are illustrated for patients aged 60 or 75 years, treated with either LRP or RARP, and with or without extraprostatic extension (EPE). The figure highlights how surgical technique, age, and pathological features may influence early continence outcomes after prostatectomy.

**Fig. 2:**
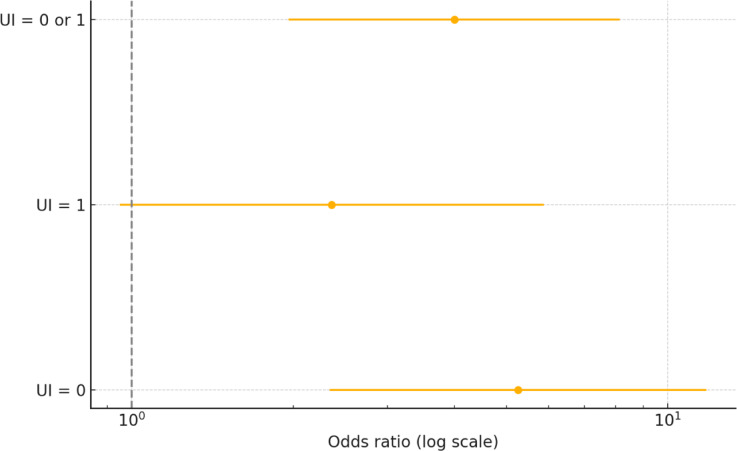
Effect of surgical approach (RARP vs LRP) on the probability of achieving urinary continence at baseline (Examination 1) using three continence definitions. Note: Adjusted odds ratios (OR) and 95% confidence intervals derived from multivariable logistic regression models (Table S2), assessing the effect of robotic surgery (RARP) compared to laparoscopic surgery (LRP) on early continence. Three definitions were applied: full continence (UI = 0), functional continence (UI = 0 or 1), and mild incontinence alone (UI = 1). RARP was associated with a more than fivefold increased odds of achieving full continence and a fourfold increase for functional continence. The association with mild incontinence (UI = 1) was weaker and did not reach statistical significance (*p* ≈ 0.065).

Descriptive statistics were used to summarize baseline clinical, pathological, and rehabilitation-related characteristics of the study population. Continuous variables were presented as means with standard deviations and compared between surgical groups using independent-samples *t*-tests. Categorical variables were summarized as counts and percentages, with between-group comparisons performed using χ^2^ tests or Fisher’s exact test, as appropriate. To identify factors associated with early urinary incontinence severity (UI stage at Examination 1), univariable and multivariable ordinal regression models were constructed. The proportional odds assumption was assessed for all ordinal regression models and was not violated. These models included clinical and pathological predictors such as age, BMI, preoperative PSA, surgical approach (RARP vs LRP), extraprostatic extension (EPE), seminal vesicle invasion (SVI), ISUP grade group, preoperative rehabilitation, and time to initiation of pelvic floor therapy. Model coefficients, 95% confidence intervals (CIs), and two-sided *p* values were reported. To further assess the effect of surgical approach on favorable continence outcomes, multivariable logistic regression models were developed for three binary endpoints: (1) full continence (UI = 0), (2) mild incontinence (UI = 1), and (3) functional continence (UI = 0 or 1). Predicted probabilities and adjusted odds ratios were visualized to illustrate the differential effect of surgical technique (Fig. 2). In addition, predicted probabilities of UI stages were calculated and visualized for representative clinical scenarios (Fig. 1), based on ordinal regression models. Ordinal regression models were also applied to continence outcomes at three-months follow-up (Examination 3), both among patients with moderate incontinence shortly after surgery (UI = 2 at Examination 1) and among those who were incontinent at baseline. The final model adjusted for baseline pad test results to account for initial incontinence severity. Exploratory time-to-event analyses using Cox proportional hazards models were performed to assess the dynamics of continence recovery in relation to surgical approach and rehabilitation timing. The starting point (*time zero*) was defined as the date of urinary catheter removal, and follow-up time was measured in days. The event was defined as the achievement of full urinary continence, corresponding to a pad test result ≤ 2 g. Patients who did not reach this endpoint during the 6-month observation period were censored at their last follow-up visit. All analyses were performed using R software (version 4.4.1; R Foundation for Statistical Computing, Vienna, Austria). Statistical significance was defined as two-sided *p* < 0.05.

## Results

The mean age of the study population was 66.2 years (SD 6.4), with no statistically significant difference between patients undergoing robot-assisted radical prostatectomy (RARP) and those treated with laparoscopic radical prostatectomy (LRP) (65.0 ± 6.7 vs. 67.0 ± 6.0 years, *p* = 0.06). Body mass index (BMI) was similar across groups (28.3 ± 3.7 kg/m^2^ overall), with no difference between RARP and LRP (*p* = 0.91). Preoperative prostate-specific antigen (PSA) tended to be lower in the RARP group (7.4 ± 3.7 ng/mL vs. 9.1 ± 8.7 ng/mL), but this difference did not reach statistical significance (*p* = 0.15). Likewise, early postoperative incontinence severity, measured by the pad test, showed a non-significant trend toward lower leakage in the RARP group (27.5 ± 44.5 g vs. 56.9 ± 86.5 g, *p* = 0.06). The mean time to initiation of pelvic floor rehabilitation was comparable between groups (37.6 ± 17.7 vs. 35.1 ± 10.5 days, *p* = 0.26).

Baseline categorical characteristics are summarized in Table 1. The distribution of key pathological variables, including extraprostatic extension (EPE), seminal vesicle invasion (SVI), and International Society of Urological Pathology grade group (ISUP), was generally balanced between groups, although a statistically significant difference was observed for ISUP grade distribution (*p* = 0.002).Urinary incontinence severity at baseline (UI stage) differed significantly between groups, with patients in the RARP group more likely to be continent or mildly incontinent (*p* < 0.001). Preoperative pelvic floor rehabilitation was initiated in the majority of patients, with no significant difference between surgical groups.

**Table 1:** Baseline categorical characteristics by surgical group (RARP vs. LRP).

Variable	Total (n = 182)	RARP (n = 76)	LRP (n = 106)	*p*-value
Rehabiltation before surgery, n (%)				
No	36 (19.8%)	18 (23.7%)	18 (17.0%)	0.3519
Yes	146 (80.2%)	58 (76.3%)	88 (83.0%)	
EPE, n (%)				
EPE 0 (none)	129 (70.9%)	60 (78.9%)	69 (65.1%)	0.1260
EPE 1 (focal)	38 (20.9%)	13 (17.1%)	25 (23.6%)	
EPE 2 (extensive)	15 (8.2%)	3 (3.9%)	12 (11.3%)	
SVI, n (%)				
No	149 (81.9%)	68 (89.5%)	81 (76.4%)	0.6823
Yes	33 (18.1%)	8 (10.5%)	25 (23.6%)	
ISUP, n (%)				
ISUP 1	27 (14.8%)	12 (15.8%)	15 (14.2%)	0.0023
ISUP 2	41 (22.5%)	17 (22.4%)	24 (22.6%)	
ISUP 3	52 (28.6%)	24 (31.6%)	28 (26.4%)	
ISUP 4	34 (18.7%)	13 (17.1%)	21 (19.8%)	
ISUP 5	28 (15.4%)	10 (13.2%)	18 (17.0%)	
Urinary incontinence (at baseline), n (%)				
Stage 0 (dry)	65 (35.7%)	33 (43.4%)	32 (30.2%)	< 0.0001
Stage 1 (mild)	19 (10.4%)	10 (13.2%)	9 (8.5%)	
Stage 2 (moderate)	58 (31.9%)	22 (28.9%)	36 (34.0%)	
Stage 3 (severe)	40 (22.0%)	11 (14.5%)	29 (27.4%)	

Distribution of key clinical and pathological categorical variables among patients treated with robot-assisted radical prostatectomy (RARP) and laparoscopic radical prostatectomy (LRP). Values are presented as number (percentage). p-values were calculated using the chi-squared test

RARP, robot-assisted radical prostatectomy; LRP, laparoscopic radical prostatectomy; EPE, extraprostatic extension; SVI, seminal vesicle invasion; ISUP, International Society of Urological Pathology grade group.

Univariable ordinal regression analysis was performed to identify factors associated with urinary incontinence (UI) severity at baseline (Examination 1), prior to the initiation of pelvic floor rehabilitation (Table S1 supplementary material). Several factors, including surgical approach, age, preoperative PSA, presence of extraprostatic extension (EPE), seminal vesicle invasion (SVI), and preoperative rehabilitation, were significantly associated with baseline UI stage. Notably, undergoing robot-assisted radical prostatectomy (RARP) was strongly associated with lower UI severity (coefficient = –1.50, 95% CI –2.08 to –0.92; *p* < 0.0001), whereas adverse pathological features such as EPE and SVI predicted higher UI stages.

In a multivariable ordinal regression model assessing predictors of UI severity at baseline (Table 2), robot-assisted radical prostatectomy (RARP) remained a strong independent factor associated with better continence (coefficient = –1.46, 95% CI –2.06 to –0.87, *p* < 0.0001). Although age and focal EPE showed borderline associations with higher UI stage, they did not reach statistical significance. Preoperative rehabilitation was also associated with improved continence outcomes, but the effect narrowly missed statistical significance (*p* = 0.073). PSA level, extensive EPE, and SVI were not significantly associated with baseline continence status in this model.

**Table 2:** Ordinal regression model assessing independent predictors of UI stage at baseline.

Variable	Coefficient	95% CI	p-value
Age (years)	0.03	(− 0.01, 0.08)	0.1263
PSA before surgery (ng/mL)	0.01	(− 0.03, 0.04)	0.6903
Type of surgery (RARP vs. LRP)	− 1.46	(− 2.06, − 0.87)	0.0000
Rehabilitation before surgery (Yes vs. No)	− 0.66	(− 1.38, 0.06)	0.0733
EPE (Focal vs. None)	0.64	(− 0.10, 1.38)	0.0899
EPE (Extensive vs. None)	0.32	(− 1.39, 2.03)	0.7142
SVI (Yes vs. No)	− 0.34	(-1.41, 0.73)	0.5330

Multivariable ordinal regression model including clinical and pathological variables to assess their independent association with urinary incontinence (UI) severity at baseline (Examination 1). Coefficients are presented with 95% confidence intervals (CI) and p-values. Negative coefficients indicate association with lower UI stage (better continence), while positive values indicate association with higher UI stage (worse continence). Reference categories: LRP for surgical approach; no EPE; no SVI; ISUP grade 1; no preoperative rehabilitation.

UI, urinary incontinence; RARP, robot-assisted radical prostatectomy; LRP, laparoscopic radical prostatectomy; PSA, prostate-specific antigen; EPE, extraprostatic extension; SVI, seminal vesicle invasion.

To better illustrate the combined effect of key predictors, predicted probabilities for each UI stage at baseline were calculated and visualized. Figure 1 shows the estimated likelihood of each UI stage depending on surgical approach (RARP vs. LRP), patient age (60 vs. 75 years), and the presence of extraprostatic extension (EPE). The figure demonstrates that patients undergoing RARP particularly younger individuals without EPE had markedly higher probabilities of achieving lower UI stages, indicating better early continence, compared to their LRP counterparts and those with adverse pathology.

These predicted probabilities, previously visualized in Fig. 1, are summarized numerically in Table 3. Younger patients without EPE undergoing RARP have the highest likelihood of full continence at baseline (UI = 0; 50.0%), whereas older patients with EPE treated with LRP exhibit the greatest probability of severe incontinence (UI = 3; 59.3%). Across all clinical profiles, RARP consistently shifted the predicted UI stage distribution toward more favorable continence outcomes.

**Table 3:** Predicted probabilities of urinary incontinence (UI) stage at baseline based on clinical scenarios (ordinal regression model).

Profile	UI 0 (dry)	UI 1 (mild)	UI 2 (moderate)	UI 3 (severe)
Age 60, LRP, no EPE	18.8%	11.5%	30.7%	39.0%
Age 60, RARP, no EPE	50.0%	15.2%	21.8%	12.9%
Age 75, LRP, with EPE	9.3%	6.8%	24.7%	59.3%
Age 75, RARP, with EPE	30.6%	14.7%	29.6%	25.2%

Predicted probabilities of each urinary incontinence (UI) stage at baseline (Examination 1) for selected hypothetical patient profiles. Estimates are based on the multivariable ordinal regression model. Patients undergoing robot-assisted radical prostatectomy (RARP), particularly those without extraprostatic extension (EPE), exhibit substantially higher likelihoods of full continence (UI = 0) compared to their laparoscopic (LRP) counterparts, especially in younger age groups.

UI, urinary incontinence; RARP, robot-assisted radical prostatectomy; LRP, laparoscopic radical prostatectomy; EPE, extraprostatic extension.

To further explore the clinical relevance of baseline continence status, we conducted multivariable logistic regression analyses predicting full continence (UI = 0), mild incontinence (UI = 1), and functional continence (UI = 0 or 1) at baseline. As shown in Table S2 (Supplementary Material), robot-assisted radical prostatectomy (RARP) was independently associated with significantly higher odds of achieving both full continence (OR = 5.26, 95% CI 2.34–11.80; *p* < 0.0001) and functional continence (defined as UI stage 0–1; OR = 4.00, 95% CI 1.96–8.14; *p* < 0.0001). The association with mild incontinence was weaker and did not reach statistical significance (*p* = 0.0651). Other clinical factors, including age, BMI, PSA, extraprostatic extension (EPE), seminal vesicle invasion (SVI), and ISUP grade group, were not consistently associated with continence outcomes in these models.

To visualize the independent effect of surgical approach on continence outcomes, adjusted odds ratios (ORs) for RARP versus LRP were plotted across three binary definitions of continence (Fig. 2). These models correspond to the multivariable logistic regression results presented in Table S2 (Supplementary Material). RARP was associated with significantly increased odds of achieving full continence (UI = 0; OR > 5, *p* < 0.0001) and functional continence (UI = 0 or 1; OR ≈ 4, *p* < 0.0001). The association with mild incontinence alone (UI = 1) was weaker and did not reach statistical significance (*p* ≈ 0.065). These findings suggest a strong beneficial effect of RARP on early postoperative continence, particularly in enabling patients to achieve dry or near-dry status shortly after surgery.

In patients who had UI stage 2 shortly after surgery, ordinal regression analysis was used to assess factors associated with urinary continence recovery at 3 months (Table 4). Among predictors, the type of surgery was strongly associated with outcome, with RARP significantly increasing the likelihood of better continence (OR = 0.21, 95% CI 0.10–0.43; *p* < 0.0001). Older age was also linked to poorer recovery (OR = 1.06, 95% CI 1.01–1.13; *p* = 0.027). Other variables, including BMI, PSA before surgery, time to rehabilitation, and pathological features such as EPE, SVI, and ISUP grade, were not significantly associated with continence outcomes. Importantly, this apparent effect of surgical approach was substantially attenuated after accounting for baseline incontinence severity, suggesting that surgical technique primarily influences the initial continence status rather than the subsequent rehabilitation-driven recovery.

**Table 4:** Predictors of urinary incontinence outcomes at 3 months in patients with UI stage 2 after surgery (ordinal regression analysis).

Variable	OR	95% CI	*p*-value
Age (years)	1.06	(1.01, 1.13)	0.0272
BMI (kg/m^2^)	1.02	(0.92, 1.12)	0.7248
Time to rehabilitation (days)	1.01	(0.99, 1.03)	0.4386
PSA before surgery (ng/mL)	0.99	(0.95, 1.04)	0.7769
Type of surgery (RARP vs. LRP)	0.21	(0.10, 0.43)	< 0.0001
Rehabilitation before surgery (Yes vs. No)	0.86	(0.39, 1.91)	0.7156
EPE (Focal vs. None)	1.73	(0.74, 4.06)	0.2072
EPE (Extensive vs. None)	2.98	(0.97, 9.19)	0.0576
SVI (Yes vs. No)	1.64	(0.69, 3.88)	0.2633
ISUP 2 vs. ISUP 1	0.86	(0.25, 2.91)	0.8075
ISUP 3 vs. ISUP 1	0.70	(0.25, 1.94)	0.4885
ISUP 4 vs. ISUP 1	0.70	(0.23, 2.13)	0.5280
ISUP 5 vs. ISUP 1	0.60	(0.19, 1.92)	0.3926

Ordinal regression model examining clinical and pathological factors associated with urinary continence (UI) status at follow-up (Examination 3) among patients who initially presented with UI stage 2 in the early postoperative period (Examination 1). Results are expressed as odds ratios (OR) with 95% confidence intervals (CI). Lower OR values indicate a higher likelihood of continence improvement. Reference categories: LRP for surgical approach; ISUP grade 1; no preoperative rehabilitation; no EPE; no SVI.

UI, urinary incontinence; OR, odds ratio; CI, confidence interval; BMI, body mass index; PSA, prostate-specific antigen; RARP, robot-assisted radical prostatectomy; LRP, laparoscopic radical prostatectomy; EPE, extraprostatic extension; SVI, seminal vesicle invasion; ISUP, International Society of Urological Pathology grade group.

Among patients who were incontinent at baseline, a final ordinal regression analysis was conducted to identify predictors of urinary continence at 3-month follow-up (Table 5). After adjusting for baseline incontinence severity using the pad test result, both delayed initiation of rehabilitation and higher initial leakage were significantly associated with worse continence outcomes. Although robotic surgery (RARP)tended to favor better recovery, its effect was no longer statistically significant in the fully adjusted model, suggesting that baseline severity may account for much of the early benefit associated with the surgical technique.

**Table 5:** Predictors of urinary continence outcomes at 3 months after surgery in patients who presented with incontinence at baseline (final ordinal regression model).

Variable	Coefficient	95% CI	*p*-value
Pad test result (g)	0.01	(0.00, 0.01)	0.0010
Age (years)	0.05	(− 0.02, 0.12)	0.1304
BMI (kg/m^2^)	0.03	(− 0.09, 0.15)	0.6645
Time to rehabilitation (days)	0.03	(0.01, 0.05)	0.0082
PSA beforesurgery (ng/mL)	0.00	(− 0.04, 0.05)	0.8685
Type of surgery (RARP vs. LRP)	− 0.82	(− 2.00, 0.36)	0.1734
Rehabilitationbeforesurgery (Yes vs. No)	0.44	(− 0.67, 1.55)	0.4354
EPE (Focal vs. None)	0.87	(− 0.13, 1.87)	0.0881
EPE (Extensive vs. None)	− 0.16	(− 2.79, 2.46)	0.9023
SVI (Yes vs. No)	− 0.84	(− 2.31, 0.63)	0.2624
ISUP2 vs. ISUP 1	− 0.78	(− 2.69, 1.12)	0.4202
ISUP 3 vs. ISUP 1	− 0.39	(− 2.04, 1.25)	0.6392
ISUP 4 vs. ISUP 1	0.52	(− 1.20, 2.23)	0.5540
ISUP 5 vs. ISUP1	− 0.25	(− 2.29, 1.79)	0.8103

Final ordinal regression model assessing clinical and pathological predictors of urinary continence (UI) status at follow-up (Examination 3) among patients with UI present at baseline (Examination 1). Patients who were continent at baseline were excluded. The model adjusts for baseline severity of incontinence based on pad test result. Results are expressed as regression coefficients with 95% confidence intervals (CI) and *p*-values. Negative coefficients indicate a higher likelihood of continence recovery. Reference categories: LRP for surgical approach; ISUP grade 1; no preoperative rehabilitation; no EPE; no SVI.

UI, urinary incontinence; CI, confidence interval; BMI, body mass index; PSA, prostate-specific antigen; RARP, robot-assisted radical prostatectomy; LRP, laparoscopic radical prostatectomy; EPE, extraprostatic extension; SVI, seminal vesicle invasion; ISUP, International Society of Urological Pathology grade group.

Cox proportional hazards models were performed to assess predictors of continence recovery in the subgroup of patients who were incontinent at baseline. In a univariable model including only surgical approach, the type of surgery (RARP vs. LRP) was not significantly associated with time to continence recovery (HR = 1.37; 95% CI 0.85–2.20; *p* = 0.20). In the multivariable model adjusted for age, BMI, PSA before surgery, pad test result, baseline UI stage, preoperative rehabilitation, and pathological factors (ISUP grade, EPE, SVI), surgical technique remained non-significant (HR = 1.30; 95% CI 0.77–2.20; *p* = 0.33). Only baseline UI stage was significantly associated with time to continence recovery (HR = 0.50; 95% CI 0.32–0.80; *p* = 0.003). Full results are provided in Supplementary Table S3.

## Discussion

This study explored the interaction between surgical approach and pelvic floor muscle therapy in shaping urinary continence recovery after radical prostatectomy. Patients undergoing RARP demonstrated better early continence, whereas long-term outcomes were largely driven by baseline incontinence severity and the timing of rehabilitation. Importantly, after accounting for baseline incontinence severity in adjusted models, surgical approach was no longer an independent predictor of continence recovery during pelvic floor rehabilitation. Taken together, the results of the logistic, ordinal, and time-to-event models are best interpreted as reflecting a two-phase continence recovery process rather than inconsistent effects of surgical technique. RARP was significantly associated with higher rates of early continence, confirming its advantage in preserving the anatomical structures essential for urinary control. This benefit likely stems from improved visualization and precision of dissection, which allow better preservation of the neurovascular bundles, external sphincter, and bladder neck. In many centers, additional technical refinements such as the Retzius-sparing and nerve-sparing approaches further contribute to early functional recovery ^18,19^. However, these factors mainly influence the initial postoperative phase and do not necessarily determine the long-term response to rehabilitation. Therefore, the observed early continence advantage associated with RARP should be interpreted as reflecting bundled perioperative technical practices rather than the robotic platform itself. The most pronounced differences between RARP and LRP in our cohort were observed before the start of physiotherapy, while subsequent outcomes converged after standardized pelvic floor muscle training. Previous studies have reported inconsistent findings regarding continence recovery after RARP versus LRP ^20–22^, underscoring that surgeon experience and perioperative management may be as important as the surgical technique itself.

Our data indicate that RARP provides an advantage in terms of early postoperative continence status, particularly within the first month after surgery; however, this difference diminishes by three months. Therefore, the decision to initiate pelvic floor rehabilitation should depend on the patient’s early postoperative pad test results rather than the surgical approach itself ^23^. Prompt identification of urinary incontinence allows tailoring of the rehabilitation program to individual patient needs. Regardless of surgical technique, structured physiotherapy programs produced comparable medium-term outcomes, confirming that rehabilitation is the major determinant of functional recovery. From a clinical perspective, this highlights the need for standardized referral protocols and coordinated postoperative care pathways for all patients, ensuring equitable access to physiotherapy irrespective of surgical technique.

Although the effectiveness of rehabilitation depends on multiple factors, the ability to correctly activate the pelvic floor muscles during exercise remains critical. Preoperative rehabilitation enables patients to learn proper muscle control and coordination, which facilitates earlier recovery of continence. In our study, preoperative physiotherapy was associated with more favorable early continence outcomes; however, its independent effect was attenuated in fully adjusted models, indicating uncertainty regarding its standalone impact ^24,25^. Importantly, preoperative pelvic floor muscle training may also mitigate the functional disadvantage of patients undergoing non-robotic procedures, helping to narrow the early continence gap between surgical techniques. Therefore, it is reasonable to recommend that all patients scheduled for radical prostatectomy participate in pelvic floor muscle training before surgery, regardless of the planned surgical approach.

In this study, all patients received individualized physiotherapy, including biofeedback and ultrasound guidance. This approach allowed continuous monitoring of muscle activation, correction of improper technique, and greater patient motivation. Evidence suggests that individualized programs are superior to verbal instructions or printed materials ^26^. In addition to improving functional outcomes, structured physiotherapy may also enhance overall recovery and shorten hospitalization, representing an important quality indicator of postoperative care in radical prostatectomy patients.

Furthermore, we found that delayed initiation of rehabilitation, regardless of whether patients underwent LRP or RARP, was associated with worse outcomes at three months. Similar to previous reports, our findings confirm that both pre- and postoperative pelvic floor training improve short- and medium-term continence outcomes, although long-term benefits remain uncertain ^27,28^. Timely referral to physiotherapy and close postoperative follow-up are therefore essential to prevent treatment delays and to optimize functional recovery. From a health-system perspective, establishing coordinated rehabilitation pathways may help standardize care and improve efficiency.

In conclusion, RARP is associated with a more favorable early postoperative continence status compared with LRP, primarily due to better preservation of anatomic structures involved in urinary control. However, long-term outcomes appear to depend more on preoperative muscle conditioning and timely initiation of pelvic floor rehabilitation than on the surgical technique itself. These findings emphasize that high-quality functional recovery after radical prostatectomy requires integration of precise surgical technique with standardized, early, and individualized physiotherapy pathways.

## Limitations

This study has several important limitations that should be considered when interpreting the findings. First, due to its observational and non-randomized design, the potential for selection bias in the choice of surgical technique cannot be excluded. Assignment to RARP or LRP may have been influenced by surgeon preference, institutional availability of robotic systems, or clinical characteristics that were not fully captured in the analysis.

Second, although key operative variables such as bladder-neck preservation, nerve-sparing status, and posterior reconstruction were recorded, some potentially relevant intraoperative details, including the precise extent of neurovascular bundle preservation or minor modifications in technique, were not evaluated in a standardized manner. However, no Retzius-sparing procedures were performed, and the experience of all participating surgeons exceeded 100 prior radical prostatectomies in each technique, minimizing the likelihood of a learning-curve effect.

Third, because several surgeons performed the operations (two robotic and five laparoscopic), we could not adjust for clustering by surgeon, which might have influenced outcomes independently of surgical approach.

Although objective measures of urinary incontinence were applied (UI stage and pad test), some data relied on patient self-report, introducing the possibility of reporting bias. Additionally, the follow-up period was limited to six months, precluding assessment of long-term rehabilitation outcomes or sustained differences between groups. We also did not evaluate the intensity or adherence to the prescribed pelvic floor muscle training programs, both of which may influence physiotherapy effectiveness.

Finally, although the overall sample size was adequate, the number of patients in certain subgroups, particularly those treated with RARP, was relatively small, which may have limited the statistical power of some multivariable analyses, especially for late outcome measures.

## Conclusions

Robot-assisted radical prostatectomy (RARP) facilitates earlier recovery of urinary continence compared with laparoscopic radical prostatectomy (LRP), primarily due to improved preservation of anatomical structures involved in urinary control. However, long-term functional outcomes depend mainly on preoperative muscle conditioning and timely initiation of postoperative pelvic floor rehabilitation rather than on the surgical technique itself.

## Supplementary Information

Below is the link to the electronic supplementary material.

Supplementary Material 1

## Data Availability

The data supporting the findings of this study are not publicly available due to privacy and ethical restrictions. Reasonable requests for access to anonymized data may be considered by the corresponding author.

## References

[1] Ma, J. et al. Robotic-Assisted versus Laparoscopic Radical Prostatectomy for Prostate Cancer: The First Separate Systematic Review and Meta-Analysis of Randomised Controlled Trials and Non-Randomised Studies. *Int J Surg* **109**, 1350–1359. 10.1097/JS9.0000000000000193 (2023). 37070788 10.1097/JS9.0000000000000193 PMC10389430

[2] Castellan, P., Ferretti, S., Litterio, G., Marchioni, M. & Schips, L. Management of Urinary Incontinence Following Radical Prostatectomy: Challenges and Solutions. *Ther Clin Risk Manag* **19**, 43–56. 10.2147/TCRM.S283305 (2023). 36686217 10.2147/TCRM.S283305 PMC9851058

[3] Kasai, T. et al. Duration and Influencing Factors of Postoperative Urinary Incontinence after Robot-Assisted Radical Prostatectomy in a Japanese Community Hospital: A Single-Center Retrospective Cohort Study. *Int J Environ Res Public Health* **20**, 4085. 10.3390/ijerph20054085 (2023). 36901096 10.3390/ijerph20054085 PMC10001515

[4] Radoja, I. & Degmečić, D. Urinary Incontinence: Diagnostic Evaluation and First-Line Treatment. *Southeastern European Medical Journal : SEEMEDJ* **4**, 63–73. 10.26332/seemedj.v4i1.133 (2020).

[5] Pak, S., Kim, M. & Ahn, H. Changes in Health-Related Quality of Life after Radical Prostatectomy for Prostate Cancer: A Longitudinal Cohort Study in Korea. *Investig Clin Urol* **59**, 313–320. 10.4111/icu.2018.59.5.313 (2018). 30182076 10.4111/icu.2018.59.5.313 PMC6121025

[6] Gacci, M. et al. Urinary and Sexual Outcomes in Long-Term (5+ Years) Prostate Cancer Disease Free Survivors after Radical Prostatectomy. *Health Qual Life Outcomes* **7**, 94. 10.1186/1477-7525-7-94 (2009). 19912640 10.1186/1477-7525-7-94 PMC2784440

[7] Teo, J. L., Zheng, Z. & Bird, S. R. Identifying the Factors Affecting ‘Patient Engagement’ in Exercise Rehabilitation. *BMC Sports Sci Med Rehabil* **14**, 18. 10.1186/s13102-022-00407-3 (2022). 35130940 10.1186/s13102-022-00407-3 PMC8819209

[8] Strączyńska, A. et al. The Impact Of Pelvic Floor Muscle Training On Urinary Incontinence In Men After Radical Prostatectomy (RP) – A Systematic Review. *CIA* **14**, 1997–2005. 10.2147/CIA.S228222 (2019). 10.2147/CIA.S228222 PMC6858802 31814714

[9] Soto González, M. et al. Early 3-Month Treatment with Comprehensive Physical Therapy Program Restores Continence in Urinary Incontinence Patients after Radical Prostatectomy: A Randomized Controlled Trial. *Neurourol Urodyn* **39**, 1529–1537. 10.1002/nau.24389 (2020). 32442334 10.1002/nau.24389

[10] de Lira, G. H. S. et al. Effects of Perioperative Pelvic Floor Muscle Training on Early Recovery of Urinary Continence and Erectile Function in Men Undergoing Radical Prostatectomy: A Randomized Clinical Trial. *Int Braz J Urol* **45**, 1196–1203. 10.1590/S1677-5538.IBJU.2019.0238 (2019). 31808408 10.1590/S1677-5538.IBJU.2019.0238 PMC6909867

[11] Farraj, H.; Alriyalat, S. Urinary Incontinence Following Robotic-Assisted Radical Prostatectomy: A Literature Review. *Cureus 16*, e53058, 10.7759/cureus.53058. 10.7759/cureus.53058 PMC10896250 38410341

[12] Filocamo, M. T. et al. Effectiveness of Early Pelvic Floor Rehabilitation Treatment for Post-Prostatectomy Incontinence. *Eur Urol* **48**, 734–738. 10.1016/j.eururo.2005.06.004 (2005). 16002204 10.1016/j.eururo.2005.06.004

[13] Gershman, B. et al. Patient-Reported Functional Outcomes Following Open, Laparoscopic, and Robotic Assisted Radical Prostatectomy Performed by High-Volume Surgeons at High-Volume Hospitals. *Eur. Urol. Focus* **2**, 172–179. 10.1016/j.euf.2015.06.011 (2016). 28723533 10.1016/j.euf.2015.06.011

[14] Jiang, J., Li, C., Liu, H.-Y. & Zhu, Z.-Y. Relationship between Abnormal Pelvic Floor Electromyography and Obstetric Factors in Postpartum Women: A Cross-Sectional Study. *BMC Women’s Health* **24**, 239. 10.1186/s12905-024-03045-8 (2024). 38616274 10.1186/s12905-024-03045-8 PMC11017559

[15] Tienza, A.; Robles ,Jose E.; Hevia ,Mateo; Algarra ,Ruben; Diez-Caballero ,Fernando; and Pascual, J.I. Prevalence Analysis of Urinary Incontinence after Radical Prostatectomy and Influential Preoperative Factors in a Single Institution. *The Aging Male* **2018**, *21*, 24–30, 10.1080/13685538.2017.1369944. 10.1080/13685538.2017.1369944 28857655

[16] Pinkhasov, R. M. et al. Prediction of Incontinence after Robot-Assisted Radical Prostatectomy: Development and Validation of a 24-Month Incontinence Nomogram. *Cancers (Basel)* **14**, 1644. 10.3390/cancers14071644 (2022). 35406416 10.3390/cancers14071644 PMC8997126

[17] Thüroff, J. W. et al. EAU Guidelines on Urinary Incontinence. *Eur. Urol.* **59**, 387–400. 10.1016/j.eururo.2010.11.021 (2011). 21130559 10.1016/j.eururo.2010.11.021

[18] Galfano, A. et al. Beyond the Learning Curve of the Retzius-Sparing Approach for Robot-Assisted Laparoscopic Radical Prostatectomy: Oncologic and Functional Results of the First 200 Patients with ≥ 1 Year of Follow-Up. *Eur Urol* **64**, 974–980. 10.1016/j.eururo.2013.06.046 (2013). 23856036 10.1016/j.eururo.2013.06.046

[19] Asimakopoulos, A. D. et al. Retzius-Sparing versus Standard Robot-Assisted Radical Prostatectomy: A Prospective Randomized Comparison on Immediate Continence Rates. *Surg Endosc* **33**, 2187–2196. 10.1007/s00464-018-6499-z (2019). 30426256 10.1007/s00464-018-6499-z

[20] İnkaya, A. et al. Comparison of Surgical, Oncological, and Functional Outcomes of Robot-Assisted and Laparoscopic Radical Prostatectomy in Patients with Prostate Cancer. *Turk J Urol* **45**, 410–417. 10.5152/tud.2019.48457 (2019). 31603415 10.5152/tud.2019.48457 PMC6788567

[21] Kural, A. R., Obek, C. & Doganca, T. Can We Accomplish Better Oncological Results with Robot-Assisted Radical Prostatectomy?. *J Endourol* **31**, S54–S58. 10.1089/end.2016.0585 (2017). 28075160 10.1089/end.2016.0585

[22] Di Mauro, E. et al. Technical Modifications Employed in RARP to Improve Early Continence Recovery: A Literature Review. *Life* **15**, 415. 10.3390/life15030415 (2025). 40141762 10.3390/life15030415 PMC11944222

[23] Terek-Derszniak, M. et al. Pelvic Floor Rehabilitation After Prostatectomy: Baseline Severity as a Predictor of Improvement—A Prospective Cohort Study. *J. Clin. Med.* **14**, 4180. 10.3390/jcm14124180 (2025). 40565928 10.3390/jcm14124180 PMC12193967

[24] Chang, J. I., Lam, V. & Patel, M. I. Preoperative Pelvic Floor Muscle Exercise and Postprostatectomy Incontinence: A Systematic Review and Meta-Analysis. *Eur Urol* **69**, 460–467. 10.1016/j.eururo.2015.11.004 (2016). 26610857 10.1016/j.eururo.2015.11.004

[25] Goonewardene, S. S., Gillatt, D. & Persad, R. A Systematic Review of PFE Pre-Prostatectomy. *J Robot Surg* **12**, 397–400. 10.1007/s11701-018-0803-8 (2018). 29564692 10.1007/s11701-018-0803-8

[26] d’Agate, D. et al. Patient Experience and Satisfaction after Same-Day Discharge Radical Prostatectomy Using a Personalized. *Digital Perioperative Programme. World J Urol* **42**, 378. 10.1007/s00345-024-05099-7 (2024). 38888646 10.1007/s00345-024-05099-7 PMC11189318

[27] Yu, K.; Bu, F.; Jian, T.; Liu, Z.; Hu, R.; Chen, S.; Lu, J. Urinary Incontinence Rehabilitation of after Radical Prostatectomy: A Systematic Review and Network Meta-Analysis. *Front. Oncol.* **2024**, *13*, 10.3389/fonc.2023.1307434. 10.3389/fonc.2023.1307434 PMC10996052 38584666

[28] Ouchi, M. et al. Physiotherapy for Continence and Muscle Function in Prostatectomy: A Randomised Controlled Trial. *BJU Int.* **134**, 398–406. 10.1111/bju.16369 (2024). 38658057 10.1111/bju.16369

