# Supplementary material for: Effectiveness of pelvic floor rehabilitation after radical prostatectomy and continence recovery in relation to surgical technique
Source: Sci Rep. 2026 Mar 6;16:12378. doi: 10.1038/s41598-026-36972-7 (PMC13084051; doi:10.1038/s41598-026-36972-7)
Supplement: Supplementary file 1 — Supplementary Material 1 [file 41598_2026_36972_MOESM1_ESM.docx]

**Supplementary Material for:**

**„Effectiveness of pelvic floor rehabilitation after radical prostatectomy and continence recovery in relation to surgical technique”**

Table S1. Univariable ordinal regression models for predictors of urinary incontinence (UI) stage at baseline (pre-rehabilitation)

| Predictor | Variable | Coefficient | 95% CI | p-value |
| --- | --- | --- | --- | --- |
| Type of surgery | RARP | -1.50 | (-2.08, -0.92) | 0.0000 |
| Age (years) | Age (years) | 0.04 | (0.00, 0.08) | 0.0484 |
| BMI (kg/m^2^) | BMI (kg/m^2^) | -0.02 | (-0.10, 0.06) | 0.5775 |
| Rehabilitation before surgery | Yes | -0.72 | (-1.44, -0.01) | 0.0471 |
| Time to rehabilitation (days) | Time to rehabilitation (days) | 0.01 | (-0.01, 0.03) | 0.4554 |
| PSA before surgery (ng/mL) | PSA before surgery (ng/mL) | 0.06 | (0.01, 0.11) | 0.0137 |
| EPE | Focal | 0.90 | (0.10, 1.70) | 0.0279 |
|  | Extensive | 1.16 | (0.34, 1.97) | 0.0055 |
| SVI | Yes | 0.84 | (0.13, 1.55) | 0.0202 |
| ISUP | ISUP_2 | -0.02 | (-0.78, 0.75) | 0.9636 |
|  | ISUP_3 | -0.29 | (-1.04, 0.46) | 0.4490 |
|  | ISUP_4 | -0.39 | (-1.17, 0.39) | 0.3263 |
|  | ISUP_5 | -0.44 | (-1.28, 0.40) | 0.3021 |

Note: Univariable ordinal regression models assessing the association between each clinical and pathological predictor and urinary incontinence (UI) stage at baseline (prior to initiation of pelvic floor rehabilitation). Negative coefficients indicate a lower likelihood of higher UI stage (i.e., better continence). Reference categories are identical to those used in the main analyses.

Abbreviations: UI – urinary incontinence; RARP – robot-assisted radical prostatectomy; LRP – laparoscopic radical prostatectomy; BMI – body mass index; PSA – prostate-specific antigen; EPE – extraprostatic extension; SVI – seminal vesicle invasion; ISUP – International Society of Urological Pathology grade group; CI – confidence interval.

Table S2. Multivariable logistic regression models predicting full, mild, and functional continence at baseline (UI 0, UI 1, UI 0 or 1)

| Predictor | UI 0 (dry) OR [95% CI), p | UI 1 (mild) OR [95% CI), p | UI 0 or 1 (good) OR [95% CI), p |
| --- | --- | --- | --- |
| Age (years) | 0.95 (0.91, 1.00), p=0.0532 | 1.00 (0.95, 1.06), p=0.8873 | 0.97 (0.93, 1.01), p=0.1271 |
| BMI (kg/m^2^) | 0.98 (0.89, 1.07), p=0.6168 | 1.01 (0.89, 1.14), p=0.8936 | 0.99 (0.90, 1.09), p=0.8641 |
| Time to rehabilitation | 1.00 (0.98, 1.03), p=0.7640 | 1.01 (0.98, 1.04), p=0.5994 | 1.00 (0.98, 1.03), p=0.9003 |
| PSA before surgery | 0.96 (0.88, 1.04), p=0.3079 | 0.95 (0.84, 1.07), p=0.3874 | 0.96 (0.88, 1.04), p=0.3056 |
| Type of surgery (RARP vs. LRP) | 5.26 (2.34, 11.80), p<0.0001 | 2.36 (0.95, 5.87), p=0.0651 | 4.00 (1.96, 8.14), p<0.0001 |
| Rehabilitation before surgery (Yes vs. No) | 1.22 (0.53, 2.79), p=0.6414 | 1.28 (0.49, 3.31), p=0.6117 | 1.19 (0.57, 2.46), p=0.6407 |
| EPE (Focal vs. None) | 0.49 (0.20, 1.20), p=0.1188 | 0.87 (0.28, 2.71), p=0.8133 | 0.58 (0.25, 1.35), p=0.2097 |
| EPE (Extensive vs. None) | 0.25 (0.06, 1.08), p=0.0642 | 0.65 (0.12, 3.38), p=0.6111 | 0.33 (0.09, 1.23), p=0.1005 |
| SVI (Present vs. None) | 0.45 (0.16, 1.24), p=0.1231 | 0.92 (0.29, 2.96), p=0.8945 | 0.57 (0.22, 1.47), p=0.2462 |
| ISUP 2 vs. ISUP 1 | 1.06 (0.30, 3.76), p=0.9229 | 1.63 (0.36, 7.39), p=0.5260 | 1.17 (0.38, 3.61), p=0.7815 |
| ISUP 3 vs. ISUP 1 | 0.80 (0.29, 2.22), p=0.6670 | 1.70 (0.45, 6.42), p=0.4322 | 1.00 (0.39, 2.60), p=0.9967 |
| ISUP 4 vs. ISUP 1 | 0.83 (0.27, 2.52), p=0.7446 | 1.48 (0.35, 6.26), p=0.5944 | 0.99 (0.35, 2.84), p=0.9812 |
| ISUP 5 vs. ISUP 1 | 0.67 (0.20, 2.24), p=0.5185 | 1.47 (0.30, 7.30), p=0.6417 | 0.85 (0.28, 2.57), p=0.7711 |

Note: Three multivariable logistic regression models were used to evaluate independent predictors of (1) full continence (UI = 0), (2) mild incontinence (UI = 1), and (3) functional continence (UI = 0 or 1) at baseline (Examination 1). Robotic surgery (RARP) was independently associated with significantly increased odds of achieving both full and functional continence compared to laparoscopic surgery (LRP). Age and pathological features showed borderline associations. Reference categories are identical to those used in the main analyses.

Abbreviations: UI – urinary incontinence; RARP – robot-assisted radical prostatectomy; LRP – laparoscopic radical prostatectomy; BMI – body mass index; PSA – prostate-specific antigen; EPE – extraprostatic extension; SVI – seminal vesicle invasion; ISUP – International Society of Urological Pathology grade group; OR – odds ratio; CI – confidence interval.

Table S3. Multivariable Cox regression model of predictors for continence recovery (patients incontinent at baseline)

| Vatiable | HR | 95% CI | p-value |
| --- | --- | --- | --- |
| Intercept | 1.56 | (0.64, 3.81) | 0.321 |
| Type of surgery (RARP vs. LRP) | 1.3 | (0.77, 2.20) | 0.33 |
| Age (years) | 1.0 | (0.97, 1.04) | 0.872 |
| BMI (kg/m^2) | 0.97 | (0.91, 1.03) | 0.325 |
| PSA before surgery (ng/mL) | 1.02 | (0.96, 1.08) | 0.475 |
| Pad test result (g) | 1.0 | (1.00, 1.00) | 0.634 |
| UI stage at baseline | 0.5 | (0.32, 0.80) | 0.003 |
| Rehabilitation before surgery ( (Yes vs. No) | 0.73 | (0.41, 1.29) | 0.282 |
| EPE (Focal vs. None) | 1.34 | (0.76, 2.35) | 0.306 |
| EPE (Extensive vs. None) | 1.14 | (0.51, 2.57) | 0.747 |
| SVI (Yes vs. No) | 0.94 | (0.50, 1.77) | 0.862 |
| ISUP 2 vs. ISUP 1 | 1.34 | (0.57, 3.13) | 0.504 |
| ISUP 3 vs. ISUP 1 | 1.5 | (0.60, 3.73) | 0.386 |
| ISUP 4 vs. ISUP 1 | 1.48 | (0.53, 4.12) | 0.456 |
| ISUP 5 vs. ISUP 1 | 0.98 | (0.32, 2.99) | 0.972 |

Note. Cox proportional hazards model examining clinical and pathological factors associated with time to urinary continence recovery among patients who were incontinent at baseline. Results are expressed as hazard ratios (HR) with 95% confidence intervals (CI). HR values greater than 1 indicate a higher rate of continence recovery; values below 1 indicate a slower recovery. Reference categories are identical to those used in the main analyses.
Abbreviations: UI – urinary incontinence; HR – hazard ratio; CI – confidence interval; BMI – body mass index; PSA – prostate-specific antigen; RARP – robot-assisted radical prostatectomy; LRP – laparoscopic radical prostatectomy; EPE – extraprostatic extension; SVI – seminal vesicle invasion; ISUP – International Society of Urological Pathology grade group.
